# Prevention of radial artery occlusion with rivaroxaban after trans-radial access coronary procedures: The RIVARAD multicentric randomized trial

**DOI:** 10.3389/fcvm.2023.1160459

**Published:** 2023-04-21

**Authors:** Rania Hammami, Slim Abid, Jdidi Jihen, Zied Triki, Imtinene Ben Mrad, Amine Kammoun, Mehdi Slim, Marwen Kacem, Houssem Thabet, Aymen Ben Abdessalem, Souad Mallek, Selma Charfeddine, Faten Triki, Samia Ernez Hejri, Ilyes Naffeti, Hicheme Denguir, Sondos Kraeim, Leila Abid

**Affiliations:** ^1^ Cardiology Department, Hedi Chaker Hospital, Sfax, Tunisia; ^2^ University of Sfax, Sfax, Tunisia; ^3^ Faculty of Medicine, University of Sfax, Sfax, Tunisia; ^4^ Epidemiology Department, Hedi Chaker Hospital, Sfax, Tunisia; ^5^ Habib Hospital Thameur, Tunis, Tunisia; ^6^ Hôpital Sahloul, Sousse, Tunisia; ^7^ Centre Hôpital Universitaire Farhat Hached, Sousse, Tunisia; ^8^ Gabes University, Gabès, Tunisia

**Keywords:** trans-radial access, radial artery occlusion, rivaroxaban, Doppler ultrasound, hemorrhagic complications

## Abstract

**Background:**

Radial artery occlusion (RAO) remains the most frequent complication of trans-radial access. Once the radial artery is occluded, its future use as an access site for coronary procedures, or as a conduit for coronary bypass grafting or fistula for hemodialysis, will be precluded. Therefore, we aimed to assess the value of the short-term use of Rivaroxaban to prevent RAO after a trans-radial coronary procedure.

**Methods:**

This was a prospective, open-label, randomized study. The patients were randomly assigned (1:1) to one of two groups: those who received Rivaroxaban 10 mg for 7 days following the trans-radial coronary procedure (the Rivaroxaban Group) and those who received the standard treatment (the Control Group). The primary outcome was an occurrence of RAO evaluated by Doppler ultrasound at 30 days, and the secondary outcomes were hemorrhagic complications according to BARC classification.

**Results:**

We included 521 patients randomized into two Groups: the Control Group (*N* = 262) and the Rivaroxaban Group (*N* = 259). The 1-month RAO was significantly reduced in the Rivaroxaban Group as compared to the Control Group [6.9% vs. 13%; *p* = 0.011, OR = 0.5, (95% CI, 0.27–0.91)]. We noted no cases of severe bleeding events (BARC3-5). The overall incidence of minor bleeding (BARC1) was 2.3%, with no significant difference between the two groups [Rivaroxaban Group = 2.7%, Control Group = 1.9%, *p* = 0.54, OR= 1.4, (95%CI 0.44–4.5)].

**Conclusions:**

Short-term postoperative anticoagulation with Rivaroxaban 10 mg for seven days reduces the rate of 1-month RAO.

## Background

Trans-radial access (TRA) has rapidly emerged as the preferred vascular access site for coronary angiography and percutaneous coronary intervention (PCI). Most of all coronary procedures are performed *via* TRA (1, 2). There are several advantages to this approach, including patient comfort, rapid hemostasis, a decrease in hospitalization stay, major adverse cardiovascular events, major bleeding, and vascular complications (3–6). However, patients are discharged without control of the patency of the radial artery in 30 to 40% of cases, and the use of doppler examination or plethysmography does not exceed 10% when control is performed (1, 7, 8). Therefore, radial artery occlusion (RAO) remains the most frequent complication of this approach and is largely underestimated. Once the radial artery is occluded, its future use as an access site for coronary procedures or as a conduit for coronary bypass grafting and fistula for hemodialysis will be precluded.

The incidence of RAO post-TRA ranged between 0.8% and 33% in observational and randomized trials (9–12). It depends on the timing and the method of the assessment of radial artery patency. Many studies showed that radial pulse is a poor indicator of radial artery patency and could still be palpable in 35% with RAO (13–15), given the suppliance through the palmar arch. Doppler ultrasound remains the best method to diagnose RAO, but it is underused (1, 7, 8). Moreover, RAO could happen without any symptoms, and the radial artery could be recanalized a few days after the procedure. However, RAO may be painful in up to 58% of patients (15) Many practices have been assessed to prevent RAO; the role of adequate procedural anticoagulation has been well established (9, 16). In contrast, the effect of postprocedural anticoagulation lacks thorough investigation. Therefore, we aimed to assess the value of a prophylactic dose of Rivaroxaban to prevent RAO after the TRA coronary procedure. Should this study prove to be positive, this could impact our routine standard of care, and it could be a new strategy to reduce the rate of RAO.

## Method

### Study design

The RIVARAD study was an interventional, prospective, open-label, randomized clinical trial aiming to evaluate the value of a prophylactic dose of Rivaroxaban in patients who underwent a trans-radial coronary procedure performed to prevent RAO. All the operators involved in the study have a large experience with the radial approach (at least 5 years).

Eligible patients were randomized, using a computer-generated randomization sequence, into two Groups: *The Rivaroxaban Group:* receiving 10 mg of Rivaroxaban for 7 days after the procedure, and *the Control Group:* receiving the standard of care treatment (i.e., no Rivaroxaban).

### Place and period

The study was multicentric; it took place in 5 Tunisian hospitals during the period between November 2021 and March 2022. The investigators were fellow physicians working in the cath labs of the five centers during the period of the study who participated in the procedures administered to the patients.

### Study population and eligibility criteria

We included patients who underwent a coronary procedure by a TRA during the period of the study.

### The inclusion criteria

We included patients aged between 18 years and 80 years who underwent a coronary procedure (coronary angiography or PCI) by TRA and provided written informed consent.

### The exclusion criteria

We did not include patients with at least one of the following criteria: administration of thrombolytic therapy in the 24 h preceding the coronary procedure, use of Gp IIb/IIIa antagonists during PCI, failure of radial artery access, access or attempted access at a second site—including contralateral radial artery, brachial, or femoral artery or vein; a palpable hematoma or clinical concern of hemostasis at the trans-radial access site after the procedure; a planned staged intervention (PCI, CABG, or noncardiac surgery) within 30 days; contraindication or high risk of bleeding with anticoagulation (bleeding requiring medical attention in the previous 6 months, thrombocytopenia (platelets < 100 × 109/L), prior intracranial hemorrhage, use of non-steroidal anti-inflammatory medications, ischemic stroke or transient ischemic attack diagnosed in the last 3 months, cardiogenic shock, chronic kidney disease with creatinine clearance of less than 30 ml/min (calculated with Cockcroft formula), liver dysfunction (Child-Pugh class B or C), a hemoglobin level below 100 g/L, history of medication noncompliance or risk factor for noncompliance, active malignancy, allergy to Rivaroxaban, patients with weight below 50 Kg, indication for curative anticoagulation (atrial fibrillation, thromboembolic events, intra-cardiac thrombus…), use of potent anti-P2 Y12 after the procedure (Ticagrelor, Prasugrel), life expectancy <30 days, history of anti-phospholipid antibody syndrome, and patients who refused to participate in the trial.

We excluded patients who returned for ultrasound control after 30 days, as well as those who underwent a new coronary procedure within 30 days and received a new dose of heparin. We called all the patients two days before the Ultrasound examination to insist on the appointment.

### TRA procedure

Coronary procedures *via* the radial artery were performed using standard techniques and equipment at the discretion of the physicians. We used Brilliant™ system (from LEPU), composed of a hydrophilic coated radial sheath, an introducer (length: 11 cm), and a needle (18 G). After local anesthesia with xylocaine 2%, we punctured the radial artery according to the Seldinger technique. An intra-arterial bolus of 200 µg nitroglycerin and 2.5 mg verapamil was routinely administered to prevent arterial spasms. For diagnostic angiography, unfractionated heparin of 3,000 IU was administrated in the cocktail at the beginning of the procedure, whereas for PCI, a total of 70–100 IU/kg was given, depending on the time of the procedure (70 IU/kg if less than 60 min, 100 IU/kg if longer than 60 min). In the case of *ad hoc* PCI, whenever the patient was not already on Aspirin, we administered 250 mg of salicylic acid intravenously. After completion of the procedure, the introducer sheath was removed, and a band compression was applied at the puncture site. The compression was gradually unscrewed every hour until hemostasis was achieved. The day after the procedure, the fellow doctor examined the patient to exclude local complications (hematoma, hemorrhagic, aneurysm.) and completed the case report form (CRF). Patients were 1:1 randomized into the Rivaroxaban Group or the Control Group.

### The case report form

For every patient, we collected data about medical history (cardiovascular risk factors, history of coronary disease, hemorrhagic complications, and history of a previous radial puncture). We also collected information about the body mass index, the results of clinical inspection and palpation, and laboratory tests (hemoglobin, platelets, creatinine clearance calculated with Cockcroft formula, anemia was defined as a hemoglobin level < 13 g/dl in males and 12 g/dl in females according to the World Health Organization). We analyzed the following procedural data: number of punctures to achieve radial access, spasm, loop during the intervention, heparin dose during the procedure, size of the sheath, type of the procedure, hemostasis time, and medications at discharge. we defined the duration of the procedure as the time from the placement of the sheath in the radial artery to the removal of the sheath from the radial artery.

### Clinical trial process

Participants of the Rivaroxaban Group received Rivaroxaban 10 mg once daily for 7 days, beginning the day after the procedure. Participants of the Control Group received their standard treatment. All patients were reexamined at 30 days by the attending physicians. They assessed the radial pulse, the observance of drugs, and the occurrence of hemorrhagic complications. On the same day, the patient underwent a doppler ultrasound exam.

### Ultrasound exam

At 30 days, all participants underwent a doppler ultrasound to assess the radial artery patency/occlusion using a Color Doppler evaluation with a 3–10—MHZ linear transducer (GE 9L-D Linear Transducer). The ultrasound examination was performed by experienced sonographers who were blinded to the trial Group. The transducer was placed parallel to the major axis of the radial artery. The color parameter settings included a low-wall filter and a low-velocity scale. The Doppler angle was maintained between 30° and 60° during the examination. The operator measured the inner diameter of the radial artery (1 cm above the level of the radial styloid). The radial artery was considered occluded if there is no antegrade flow signal.

### Ethical considerations

This study was authorized on 30/09/2021 by the “C.P.P.SUD” committee (the committee of personal protection in the south of Tunisia) with the following approval number: 348/2021.

Before inclusion in this study, each participant gave written consent.

The clinical trial was registered on the Pan African Clinical Trial Registry under the following number: PACTR202110633346282.

### The study outcomes

The primary endpoint consisted of the rate of radial artery occlusion at 30 days, assessed by ultrasound examination of the wrist.

The secondary endpoints consisted of the incidence of hemorrhagic events at 30 days (defined according to the Bleeding Academic Research Consortium (BARC) criteria and classification (17)), local complications on the puncture point (aneurysm, hematoma, arterio-venous fistula).

### Statistics

Given the lack of similar studies in the literature when establishing the protocol, we carried out a pre-survey on 15 patients for each group to determine the number of subjects needed. RAO occurred in 1 patient/15 in the Rivaroxaban Group (6.6%) and in 2 patients/15 in the Control Group (13.3%). The number of patients that needed to be included was calculated by considering the previous incidences. For a risk of error *α *= 5% and a power *β *= 80%, we needed to include at least 256 patients in each group. We increased the number included in each group by 5%. The Statistical analyses were performed using the IBM SPSS Statistics v.23 software.

Categorical variables were compared using the Chi-square or Fisher exact test when required. Continuous variables were compared with the unpaired Student's *t*-test or the Mann–Whitney U test if normally or not normally distributed, respectively.

We used univariate and multivariate logistic regression analysis models to identify independent predictors for RAO, with results presented as odds ratio (OR) with a 95% confidence interval (CI). Variables with *P* < 0.20 in the univariate analysis or thought to be clinically important (randomized allocation) were included in the multivariate model. Statistical significance was defined by a *p*-value < 0.05.

## Results

### Patient characteristics

**Figure 1:**
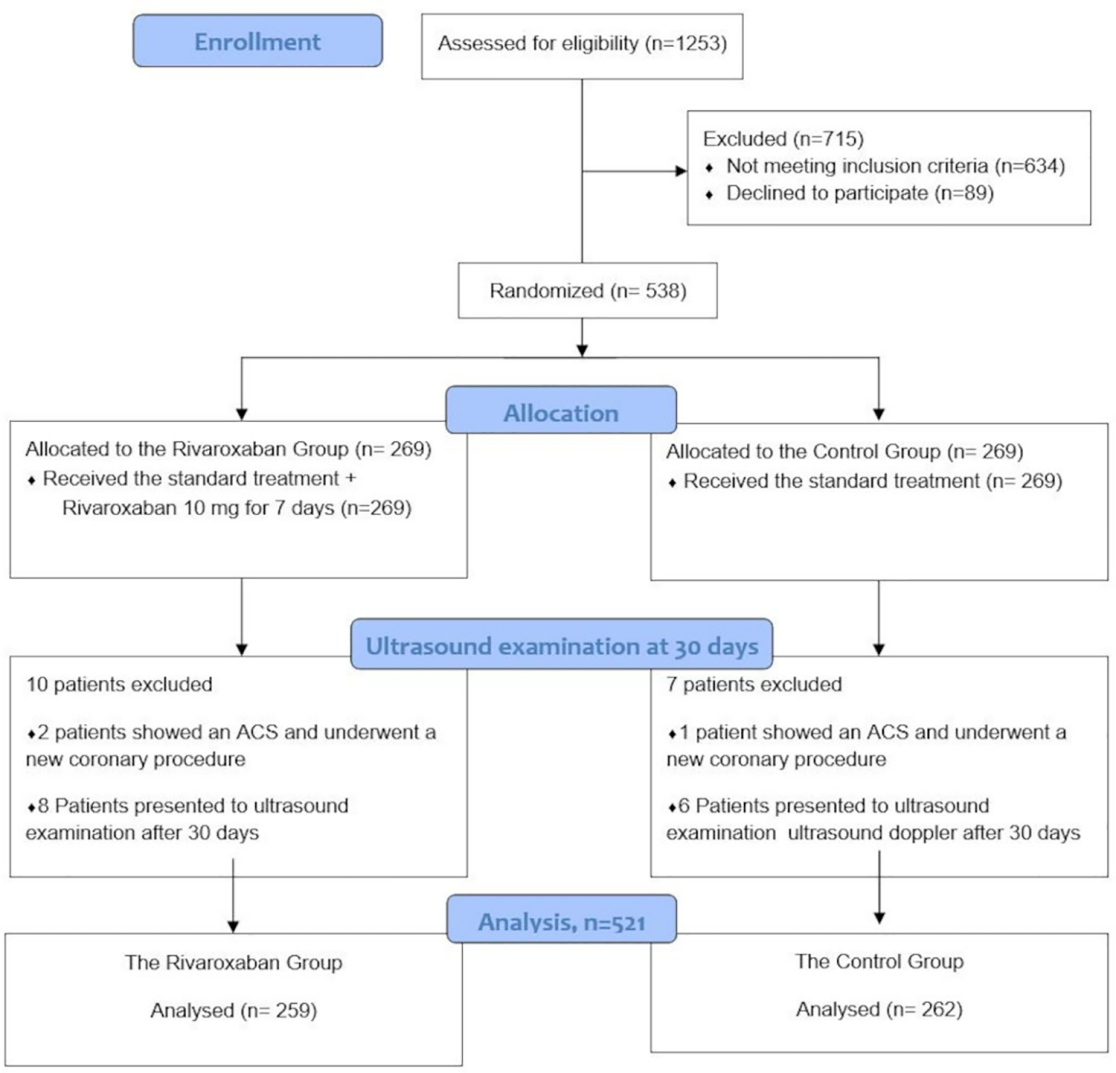
Flow Diagram of the RIVARAD.

A total of 1,253 consecutive patients who underwent a coronary procedure in the five centers were screened for inclusion in the RIVARAD trial between November 2021 and March 2022. Of these patients, 538 patients were eligible for the study. They gave their consent and were randomized into the Rivaroxaban Group and a Control Group. Seventeen patients were excluded from the study: 14 patients presented for the ultrasound examination later than 30 days, and three patients showed acute coronary syndrome within 30 days and underwent a new coronary procedure. Finally, we analyzed the outcomes in 521 patients (262 in the Control Group and 259 patients in the Rivaroxaban Group (Figure 1). The mean age of our patients was 59.7 ± 9.8 years. Most of them were male (67.6%). The prevalence of different cardiovascular risk factors was high: diabetes (48.2%), smoking (48%), hypertension (54.5%), and dyslipidemia (39.5%). A percutaneous coronary intervention was performed in 35.1% of the patients. Baseline demographic data, medical history, procedural characteristics, laboratory results, and medications were similar between the 2 Groups and were summarized in Tables 1, 2.

**Table 1:** Baseline demographics and clinical characteristics.

Parameter	Control group	Rivaroxaban group	*P* value
	(*n* = 262)	(*n* = 259)	
Clinical characteristics			
Age, y	60.1 ± 9	59.41 ± 10	0.410
Weight, Kg	79.04 ± 11.3	78.7 ± 12.6	0.79
BMI, Kg/m^2^	27.9 ± 4.47	27.1 ± 4.6	0.055
Male, *n* (%)	167 (63.7)	185 (71.4)	0.060
Current Smoking, *n* (%)	115 (43.9)	135 (52.1)	0.060
Hypertension, *n* (%)	147 (56.1)	137 (52.9)	0.462
Diabetes, *n* (%)	137 (52.3)	114 (44)	0.058
Hyperlipidemia, *n* (%)	96 (36.6)	110 (42.5)	0.17
Anemia, *n* (%)	97 (37)	82 (31.7)	0.19
Renal failure, *n* (%)	49 (18.7)	38 (14.7)	0.21
Previous trans-radial procedures, *n* (%)	58 (22.1)	76 (29.3)	0.06
Known Peripheral arterial disease, *n* (%)	24 (9.2)	14 (5.4)	0.09
Carotid atheroma, *n* (%)	14 (5.3)	6 (2.3)	0.07
Coronary history disease, *n* (%)	38 (14.5)	45 (17.4)	0.37
Indication of the coronary procedure			
Acute coronary syndrome, *n* (%)	176 (67.2)	190 (73.4)	0.12
STEMI, *n* (%)	23 (8.8)	27 (10.4)	0.52
NSTE- ACS, positive Troponin, *n* (%)	58 (22.1)	58 (22.4)	0.94
NSTE- ACS, negative Troponin	95 (36.3)	105 (40.5)	0.31
Chronic coronary syndrome, *n* (%)	70 (26.7)	58 (22.4)	0.25
Left ventricle dysfunction, *n* (%)	11 (4.2)	4 (1.5)	0.11
Arrhythmia, *n* (%)	2 (0.8)	6 (2.3)	0.17
Valvular diseases, *n* (%)	3 (1.1)	1 (0.4)	1
Laboratory data			
Creatinine clearance, ml/min	92.7 ± 26.3	92.2 ± 25.3	0.82
Hemoglobin, g/dl	13.23 ± 1.4	13.47 ± 1.4	0.056
Platelet, 10^9^/L	241.22 ± 68.9	229.40 ± 66.65	0.052
Medications after the procedure			
Aspirin, *n* (%)	118 (45.0)	96 (37.1)	0.06
DAPT (clopidogrel + Aspirin), *n* (%)	120 (45.8)	125 (48.3)	0.57
Statin, *n* (%)	112 (42.7)	116 (44.8)	0.63
No antiplatelets treatments	24 (9.2)	38 (14.7)	0.052

BMI, Body mass index; STEMI, ST-elevation myocardial infarction; NSTE- ACS, non-ST elevation acute coronary syndrome; DAPT, Dual antiplatelet treatment.

**Table 2:** Procedural data.

	Control group	Rivaroxaban group	*P* value
	(*n *= 262)	(*n *= 259)	
PCI, *n* (%)	85 (32.4)	98 (37.8)	0.19
Sheath diameter			
5F Sheath, *n* (%)	98 (37.4)	79 (30.5)	0.096
6F Sheath, *n* (%)	164 (62.4)	180 (69.5)	0.096
Puncture Attempt >1, *n* (%)	117 (45)	134 (51.1)	0.17
SAP during the procedure, mmHg	131.63 ± 17.7	131.25 ± 15.6	0.79
DAP during the procedure, mmHg	72.22 ± 12.47	72.0 ± 10.76	0.83
HR during the procedure, bpm	74.41 ± 9.77	74.45 ± 10.7	0.97
Puncture attempts number	1.57 ± 0.63	1.50 ± 0.60	0.22
Procedure duration, min	22.89 ± 11.3	24.09 ± 15.2	0.3
Heparin, IU	5190 ± 1679	5337 ± 1946	0.35
acetylsalicylic acid IV injection, *n* (%)	18 (6.9%	13 (5%)	0.37
Radial spasm, *n* (%)	19 (7.3)	20 (7.7)	0.83
Radial loop, *n* (%)	39 (14.9)	42 (16.2)	0.67
Hemostatic time >6 h, *n* (%)	182 (69.5)	169 (65.3)	0.30
Outer Radial artery lumen diameter, mm	2.32 ± 0.47	2.35 ± 0.43	0.21

DAP, diastolic arterial pressure; HR, Heart rate; PCI, percutaneous coronary intervention; SAP, systolic arterial pressure.

### End points and complications

**Figure 2:**
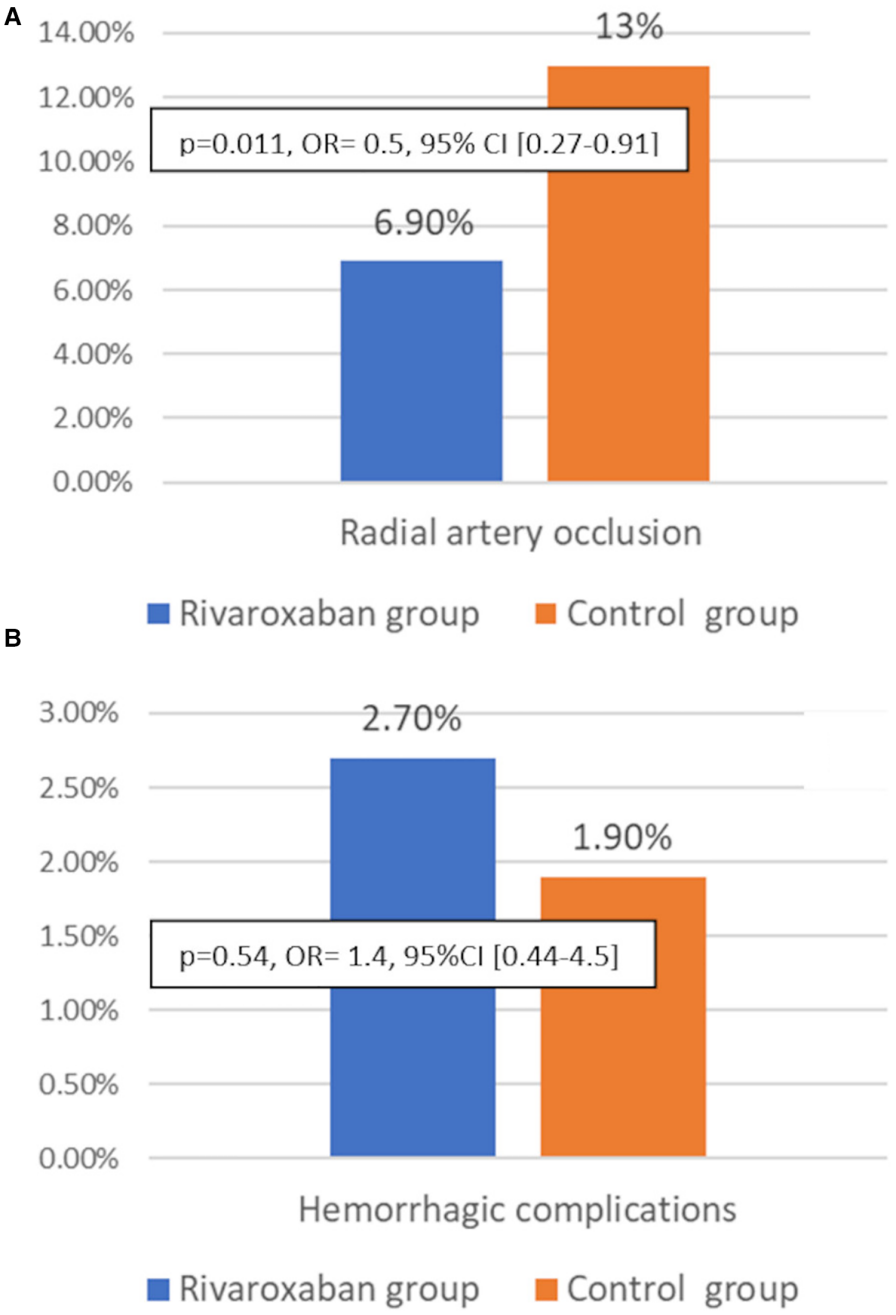
Incidence of radial artery occlusion (**A**) and hemorrhagic complications at 30 days in the Rivaroxaban group (blue bars) and the Control group (orange bars) (**B**).

The primary endpoint: The overall incidence of RAO at 30 days was 10%. RAO occurred in 34 patients of the Control Group and in 18 patients of the Rivaroxaban Group and was significantly lower in the Rivaroxaban Group compared with the Control Group [13% vs. 6.9%; *p* = 0.011, OR =  0.5, 95% CI (0.27–0.91)] (Figure 2). To avoid RAO in one patient, about 16 patients had to be treated with daily Rivaroxaban (10 mg). All cases of RAO were asymptomatic. No patient experienced radial artery pseudoaneurysm, local hematoma, arteriovenous fistula, or compartment syndrome.

On physical examination, the radial pulse was not palpable at 30 days in 32 patients in the Control Group and in 15 patients in the Rivaroxaban Group (12.2% vs. 5.8%, *p* = 0.011). There was a discrepancy between clinical examination findings and ultrasound findings in 19 patients (3.6%). In 12 patients, we were able to palpate a radial pulse, but the doppler ultrasound showed an occlusion of the artery, while in 7 patients, we did not find a radial pulse even though the artery was patent on ultrasound examination.

We noted 12 cases (2.3%) of minor bleeding (BARC 1) (epistaxis (*n* = 7), gingivorrhagia (*n* = 2), hemoptotic sputum = 3), and there were no significant differences between the 2 Groups [Rivaroxaban Group = 2.7%, Control Group = 1.9%, *p* = 0.54, OR =  1.4, 95% CI (0.44–4.5)]. Minor bleeding occurred in four patients treated with DAPT + Rivaroxaban, three patients treated with Aspirin + Rivaroxaban, three patients treated with DAPT, and two patients treated with Aspirin. We did not observe any hemorrhagic complications in patients treated with Rivaroxaban alone.

### Predictors of 30-day radial artery occlusion

Logistic regression analysis was performed to identify predictors for 1-month RAO (Table 3).

**Table 3:** Predictors of 30 days’ RAO.

	Univariate logistic regression			Multivariate logistic regression		
	OR	95% CI	*P* Value	OR	95% CI	*P* Value
Age	0.96	0.93–0.98	0.004			
Female	1.9	1.07–3.141	0.02	3.71	1.74–7.9	0.001
BMI	1.03	0.97–1.09	0.29			
Current smoking	1.84	1.02–3.31	0.03	3.96	1.85–8.49	<10^−3^
Hypertension	1.5	0.83–2.72	0.17			
Diabetes	1.08	0.61–1.9	0.78			
Hyperlipidemia	1.35	0.76–2.4	0.3			
Renal failure	2.49	1.31–4.72	0.004			
Previous Trans radial procedures	1.77	0.97–3.23	0.06	2.07	1.07–3.98	0.029
Hemostatic Time > 6 Hours	2.17	1.06–4.44	0.03			
ACS	0.5	0.27–0.91	0.022			
PCI	0.72	0.38–1.36	0.31			
Puncture attempts >1	1.8	1.01–3.28	0.04			
Sheath Type						
Sheath 5 F	0.85	0.45–1.57	0.6			
Heparin dosage	0.98	0.96–1.001	0.06	0.98	0.96–1	0.047
Procedure duration, min	0.99	0.96–1.01	0.57			
Creatinine, μmol/L	1.005	0.99–1.01	0.34			
Hemoglobin, g/dl	1.03	0.84–1.26	0.74			
Platelet, 10^9^/L	1.001	0.99–1.005	0.61			
Statin use	0.78	0.43–1.41	0.41			
Aspirin use	1.25	0.7–2.23	0.43			
DAPT	0.56	0.31–1.02	0.059			
Rivaroxaban use	0.5	0.27–0.91	0.02	0.47	0.25–087	0.018

BMI, Body mass index; ACS, Acute coronary syndrome; PCI, Percutaneous coronary Intervention; DAPT, dual Antiplatelet treatment.

In multivariate logistic regression analysis models, female sex [OR, 3.7 (95% CI, 1.7–7.9); *P* = 0.001], Current Smoking [OR, 3.9 (95% CI, 1.85–8.79); *P *< 0.001] and history of previous trans-radial puncture [OR, 2.07(95% CI, 1.07–3.98); *P *< 0.029] were identified as independent predictors of 30-day RAOs. In our trial, the incidence of RAO at 1 month in smoking women with a history of previous trans-radial puncture was 50%.

Rivaroxaban use [OR, 0.47 (95% CI, 0.25–0.87); *P* = 0.018] and per-procedural heparin dosage [OR, 0.98, (95% CI, 0.96–0.99); *P* = 0.047] were shown to be an independent protective factor against 1-month RAO.

## Discussion

Radial artery access has been widely used given the low incidence of local and general complications; however, there are low rates of adherence to practices that are known to improve outcomes (1, 7, 8). There is no standard protocol for radial band deflation, and it varies across institutions. In a recent survey, the authors noted that more than 30% of operators do not assess radial artery patency before discharge, and most of them do not believe that patent hemostasis is necessary. Besides, half of the operators in the USA and more than 90% of operators out of the USA never use ultrasound to assess radial artery patency (7). Indeed, RAO remains a common and largely underestimated complication of TRA. In recent metanalyses, adequate procedural anticoagulation has been identified as the strongest protector against RAO occurrence (16, 18) without compromising safety. However, the effect of postprocedural anticoagulation has not been well investigated. To our knowledge, the RIVARAD is the first multicentric randomized trial, with 30-day RAOs as the primary endpoint, that proved the efficacy of short-term use of Rivaroxaban to prevent RAO in the real-world Radial approach daily practice. Recently, the RESTORE, a Chinese trial with 24-hour RAOs as the primary endpoint and 30-day RAOs as the secondary endpoint, concluded that Rivaroxaban did not show any benefit at 24 h but could be efficient at 30 days to prevent RAO. In this study, unfractionated heparin of 2,500 IU was given for diagnostic angiography, and in total, 100 IU/kg was given for *ad hoc* intravascular ultrasounds or PCI (19). Indeed, the RESTORE trial was a single-center trial that assessed the impact of short-term use of Rivaroxaban (10 mg daily for 7 days) to prevent RAO in 382 patients. All the procedures were performed using a 6 F sheath, and all the patients were pretreated with DAPT. The authors did not find any significant difference between the Rivaroxaban Group and the Control Group for the primary endpoint (RAO at 24 Hours: 8.9% vs. 11.5%; *P* = 0.398). Only 314 patients underwent doppler examination at 30 days. The 1-month RAO was significantly reduced in the Rivaroxaban Group compared with the placebo Group [3.8% vs. 11.5%; *P* = 0.011, OR, 0.22 (95% CI, 0.08–0.65); *P* = 0.006] (19). In our trial, we found it more convenient to fix primary endpoints as RAO at 30 days after the intervention and not early at 24 Hours; however, the prevalence of the impalpable radial pulse was similar in the two groups at 24 Hours. Moreover, we excluded patients who showed acute coronary syndrome within 30 days and underwent a new coronary procedure because they would receive heparin once again.

We have chosen Rivaroxaban because it is a single-intake direct oral anticoagulant and not only because of its primary effect on coagulation but also because of its increasingly proven effect in the improvement of endothelial function (20). Indeed, the endothelial injury of the radial artery, the subsequent decrease in blood flow, and the negative remodeling of the arterial wall have been identified as the main mechanisms of RAO (5). Based on its shown effectiveness and tolerability for the prevention of venous thromboembolism, Rivaroxaban 10 mg daily was chosen for this investigation.

In the RIVARAD, the overall incidence of RAO was 10% and was significantly lower in the Rivaroxaban Group [13% vs. 6.9%; *p* = 0.011, OR = 0.5 95% CI (0.27–0.91)]. The incidence of this complication after a TRA procedure in north African patients has not been well identified. In a previous study from our center, comparing the feasibility and the safety of the distal radial approach and the conventional radial approach, we reported an incidence of 3.1% at 24 Hours in the conventional Group (21). In that study, the incidence was lower because we excluded all patients with an impalpable radial distal pulse before the procedure, and the history of previous radial punction was less frequent compared to the RIVARAD trial (11% in the previous study vs. 25% in the RIVARAD trial). Besides, the prevalence of female sex and smokers was lower in the previous study than in the RIVARAD trial. The high incidence reflects real-world daily practice; patent hemostasis technique and ipsilateral ulnar compression are, unfortunately, rarely used in radial hemostasis protocols. In fact, given the issues of Trans Radial band availability in all centers during the whole period of the trial, we opted to manual compression in our trial.

The incidence of RAO in the RIVARAD trial was similar to the RESTORE trial and remains high compared to the previous randomized trials as we did not use a patent hemostasis protocol during postprocedural compression, the hemostatic time was long in the two trials, and the prevalence of history of trans-radial puncture and diabetes was high in the two trials.

Interventionists must be aware of the adequate method to assess radial artery patency and predictors of RAO to maximize the efforts toward reducing RAO rates. The diagnosis of RAO by pulse palpation is not accurate. A palpable pulse does not exclude the diagnosis of RAO because collateral blood flow may supply the periphery of the radial artery distally to the occlusion, giving a false impression of radial artery patency. In the RIVARAD trial, there was a discrepancy between the clinical data and the ultrasound examination in 19 patients at 30 days.

Many RAO predictors have been described in the literature, like female gender, older/younger age, lower body size, diabetes mellitus, longer fluoroscopy duration, a higher number of puncture attempts, non-patent hemostasis, and longer hemostasis or bandage time (21–23). The periprocedural dose of heparin remains the strongest negative predictor of RAO (16, 24, 25).

For diagnostic procedures, the dose of 5,000 UI for heparin anticoagulation was only given by 45% of operators in the Italian survey (8) and less than 60% of operators in the second international survey (7). That's why we tried to conduct this trial under the same conditions of real-life practices.

In our trial, smoking, female gender, and history of previous trans-radial punctures were found as predictors of RAO. Thus, one smoking woman out of 2 showed RAO in our cohort when she had a history of trans-radial puncture. This could be related to the smaller radial artery diameter in females compared to males, the known thrombogenic effect of tobacco, and the risk of repeated traumatism in the artery. In contrast, the administration of Rivaroxaban 10 mg daily for one week after the procedure and the per-procedural dosage of heparin were independent negative predictors of 1-month RAO on multivariate logistic regression analysis, with no excess bleeding. The RESTORE trial showed the same safety outcomes (19). In the two trials, no patient showed hemorrhagic BARC3-5 events.

In our practice**,** the number of outpatient procedures is increasing, and coronary artery disease is affecting younger patients with a growing need for repeat procedures in many patients. The RIVARAD study offers an attractive and safe solution to reduce RAO after TRA, especially in those at high risk. The currently recruiting Phase 3 trial CAPITAL-RAPTOR (Rivaroxaban Post-Trans-radial Access for the Prevention of Radial Artery Occlusion, NCT03630055), will provide answers concerning the efficacy and safety of a higher dose of Rivaroxaban (15 mg for 7 days). Further large, superiority randomized trials comparing the different doses of Rivaroxaban are needed.

## Study limitations

Our study, although multicentric and randomized, presents some limitations. Indeed, the recommendations of “Best Practices for the Radial Approach” were not well-observed, such as compression of the ipsilateral ulnar artery, the low heparin dose for diagnosis procedures, and the use of patent hemostasis, but this reflects our “hectic,” real-life daily practices. Moreover, we did not use a placebo as a comparator because of the financial constraints and the lengthy production procedure in our country (up to 2–3 years). However, our primary endpoint consists of the diagnosis of RAO on doppler examination, and there was no psychological implication in the occurrence of RAO; hence, placebo use does not affect our outcomes. The hemostasis time was also another limitation of the trial and was also relatively more prolonged than the recommendation by the international consensus. However, this trial reflects the real-world practice of hemostasis management after TRA catheterization in our centers, and the problems of availability of compression bands.

## Conclusion

The RIVARAD study was a multicentric randomized trial that showed the efficacy and the safety of short term post procedural Rivaroxaban to reduce the rate of RAO after TRA. This would be an interesting preventive measure in our real-life daily practice especially with the increase in the number of ambulatory procedures and the difficulty of achieving patent hemostasis in routine.

## Data Availability

The original contributions presented in the study are included in the article/supplementary materials, further inquiries can be directed to the corresponding author.
